# TNF-α Suppresses Apelin Receptor Expression in Mouse Quadriceps Femoris-Derived Cells

**DOI:** 10.3390/cimb44070217

**Published:** 2022-07-08

**Authors:** Tomohisa Koyama, Kentaro Uchida, Makoto Itakura, Masayuki Miyagi, Ryo Tazawa, Gen Inoue, Kensuke Fukushima, Yoshihisa Ohashi, Ayumi Tsukada, Masashi Takaso

**Affiliations:** 1 Department of Orthopedic Surgery, Kitasato University School of Medicine, 1-15-1 Minami-ku, Kitasato, Sagamihara City 252-0374, Kanagawa, Japan; tomohisakoyama1989@gmail.com (T.K.); masayuki008@aol.com (M.M.); popolo-55@hotmail.co.jp (R.T.); ginoue@kitasato-u.ac.jp (G.I.); kenfu@r4.dion.ne.jp (K.F.); 44134413oo@gmail.com (Y.O.); amidesutarere9010@yahoo.co.jp (A.T.); mtakaso@kitasato-u.ac.jp (M.T.); 2 Department of Biochemistry, Kitasato University School of Medicine, 1-15-1 Minami-ku, Kitasato, Sagamihara City 252-0374, Kanagawa, Japan; mitakura@med.kitasato-u.ac.jp

**Keywords:** apelin receptor (APJ), TNF-α, skeletal muscle, aging

## Abstract

Expression of the apelin receptor, APJ, in skeletal muscle (SM) is known to decrease with age, but the underlying mechanism remains unclear. Increased tumor necrosis factor (TNF)-α levels are observed in SM with age and are associated with muscle atrophy. To investigate the possible interconnection between TNF-α elevation and APJ reduction with aging, we investigated the effect of TNF-α on APJ expression in cells derived from the quadriceps femoris of C57BL/6J mice. Expression of *Tnfa* and *Apj* in the quadriceps femoris was compared between 4- (young) and 24-month-old (old) C57BL/6J mice (*n* = 10 each) using qPCR. Additionally, APJ-positive cells and TNF-α protein were analyzed by flow cytometry and Western blotting, respectively. Further, quadricep-derived cells were exposed to 0 (control) or 25 ng/mL TNF-α, and the effect on *Apj* expression was examined by qRT-PCR. *Apj* expression and the ratio of APJ-positive cells among quadricep cells were significantly lower in old compared to young mice. In contrast, levels of *Tnfa* mRNA and TNF-α protein were significantly elevated in old compared to young mice. Exposing young and old derived quadricep cells to TNF-α for 8 and 24 h caused *Apj* levels to significantly decrease. TNF-α suppresses APJ expression in muscle cells in vitro. The increase in TNF-α observed in SM with age may induce a decrease in APJ expression.

## 1. Introduction

Sarcopenia is a disorder that is characterized by the gradual deterioration of skeletal muscle (SM) mass and strength as a person ages, and leads to a decline in autonomy in older people [1,2]. Loss of motility and mobility is increasingly believed to be one of the most compelling indicators of poor health outcomes in older people [3,4]. Knowledge accumulated through decades of extensive research with many different animal and human models on skeletal muscle atrophy/deconditioning has provided us with a good understanding of the cellular processes implicated [5,6,7,8,9,10]. To identify other potential cellular mechanisms and improve understanding of those already discovered, is important to reveal the underlying causes of sarcopenia.

The G protein-coupled apelin receptor (APJ) is widely expressed throughout the body. APJ is implicated in various physiological processes, including energy metabolism, cardiovascular function, and angiogenesis. A recent study reported that APJ and its ligand apelin contribute to muscle metabolism and that APJ gene expression is decreased in old mice [11]. However, the cause of the decrease in APJ with age has not been fully clarified.

Interestingly, inflammatory cytokine levels are reported to be elevated in the blood and skeletal muscle tissue of sarcopenia patients [12,13,14,15,16,17,18]. Higher plasma concentrations of tumor necrosis factor (TNF)-α result in reduced muscle mass and strength in normally functioning elderly people [18]. A single intraperitoneal administration of TNF-α (100 μg/kg) to male ICR mice increased the cytoplasmic oxidative activity of muscle fibers isolated from the intercostal diaphragm and decreased the maximal force of the diaphragm [19]. A recent study reported that elevated concentrations of TNF-α in the SM of old mice are associated with atrophy [20]. Further, a strong link between TNF-α, protein turnover alteration and muscle deconditioning with aging has been reported [20,21,22,23,24,25,26]. TNF-α induced protein loss in skeletal muscle myocytes via reactive oxygen-mediated NF-κB activation [25]. TNF-α signaling induced muscle fiber-specific apoptosis [27]. Further, TNF-α upregulated Atrogin1/MAFbx, which appear to be essential for accelerated muscle protein loss [22]. Several studies have reported that APJ signaling regulates TNF-α in macrophages, adipocytes, and hepatocytes in vitro [28,29,30]. However, it remains unclear whether TNF-α regulates APJ expression in cells derived from SM.

To investigate the possible interconnection between TNF-α elevation and APJ reduction with aging, we investigated the effect of TNF-α on APJ expression in cells derived from the quadriceps femoris of C57BL/6J mice.

## 2. Materials and Methods

### 2.1. Animals

This study was conducted on male C57BL/6J mice (Jackson Laboratory Japan, Yokohama, Japan). C57BL/6J mice were housed at Jackson Laboratory Japan (Kanagawa, Japan) under a semibarrier system with controlled temperature (23 ± 2 °C), humidity (55% ± 10%), and light (12 h light/dark cycle). The study protocol was approved by the Kitasato University School of Medicine Animal Care Committee (reference number: 2021-046).

A previous study showed that muscle/body weight was reduced in 24-month-old C57BL/6 mice compare to 3-month-old C57BL/6 mice [31]. Therefore, we categorized 3-month-old mice (*n* = 10) as the “young” group and 24-month-old mice (*n* = 10) as the “old” group. Body weight (g) and muscle weight of the quadriceps femoris (mg) were measured and muscle weight (mg)/body weight (*n* = 10) was calculated. *Apelin*, *Apj* and *Tnfa* mRNA expression in the quadriceps femoris was examined using real-time PCR and compared between the two age groups. In addition, TNF-α protein expression was examined by Western blotting. To determine whether TNF-α affects apelin and APJ expression in SM, quadriceps femoris tissue from young (*n* = 5) and old mice (*n* = 5) was digested with collagenase. Muscle cells were subsequently harvested and exposed to 0 ng/mL (control: culture medium only), 2.5 ng/mL or 25 ng/mL TNF-α for 8 and 24 h. mRNA was extracted from the stimulated cells and *Apelin* and *Apj* expression was measured using real-time PCR.

### 2.2. Real-Time PCR

C57BL/6J mice were sacrificed by inhalation anesthesia with isoflurane. Using a scalpel, the skin and fascia of the upper leg were removed and the quadriceps femoris was harvested. The harvested tissue was then subjected to TRIzol (Invitrogen, Carlsbad, CA, USA) treatment to extract total RNA based on the manufacturer’s protocol. The total RNA formed the template for cDNA synthesis using SuperScript III RT (Thermo Fisher Scientific, CA, USA) in a PCR reaction that comprised cDNA, TB Green Premix Ex Taq (Takara, Kyoto, Japan) and a specific primer set. Primers in the primer set were fashioned on Primer Blast software and made by Hokkaido System Science Co., Ltd. (Sapporo, Japan). Table 1 lists the primer sequences adopted in this study. Amplified products were examined for specificity using melt curve analysis. Quantitative PCR was conducted on a CFX connect real-time PCR detection system (Bio-Rad, Hercules, CA, USA) with a denaturation step at 95 °C for 1 min, 40 cycles of 95 °C for 5 s and 60 °C for 30 s. We evaluated β-actin and GAPDH as housekeeping genes. Because β-actin gene differed between young and old mice, levels of each mRNA of interest were normalized to concentrations of GAPDH.

### 2.3. Western Blotting

Protein levels of TNF-α were measured using Western blotting. After homogenizing muscle cells in sodium dodecyl sulfate (SDS) sample buffer, the homogenates immediately heated at 95 °C for 10 min. Protein concentrations were determined by a bicinchoninic acid (BCA) assay kit (Thermo Fisher Scientific, Inc., Waltham, MA, USA). No protein degradation was confirmed in Coomassie Brilliant Blue staining. The homogenates (5 μg/lane) were subjected to SDS-polyacrylamide gel electrophoresis. The separated proteins in the gel were then electrophoretically transferred to a polyvinylidene difluoride membrane in blotting buffer. The membrane was subsequently treated with 10% skim milk in TBST for 30 min at 25 °C to prevent non-specific reactions before incubating with anti-TNF-α antibody (1:1000; catalog number. Ab6671, Abcam Cambridge, UK) or anti-GAPDH antibody (1:5000; FUJIFILM Wako Pure Chemical Co., Osaka, Japan) for 60 min at 25 °C. After further incubating with goat anti-rabbit antibody conjugated to HRP (catalog number. 211-035-109, RRID: AB_2339150, Jackson Immuno Research Laboratories; West Grove, PA, USA) for 60 min at 25 °C, the membrane was washed a final time. Protein bands were subsequently visualized using enhanced chemiluminescence (catalog number 07880, Chemi-Lumi One L; Nacalai Tesque, Kyoto, Japan) and a luminescent image analyzer with a CCD imager (LAS-4000mini; Fuji Photo Film Co., Tokyo, Japan). Relative TNF-α expression was normalized to GAPDH.

### 2.4. Flow Cytometry

Tissue samples of the quadriceps femoris taken from young and old mice (*n* = 5 each) were treated with a 20 mL solution of 0.1% collagenase (Catalog Number. 03222364, Fujifilm Wako Pure Chemical Corporation, Osaka, Japan) at 37 °C for 1 h. The digested samples were then filtered through a nylon mesh filter (pluriStrainer 100 µm, pluriSelect, Leipzig, Germany) to obtain cell suspensions. The cells were then treated with the following antibodies: anti-CD45-PE/Cy7 (Clone: 30-F11, Catalog number 103113, BioLegend, CA, USA) and anti-Sca1-APC-Cy7 (Clone: D7, Catalog number 108126, BioLegend) for 1 h at 4 °C. CD45 is a marker for pan-hematopoietic cells and Sca1 is a marker for mature myocytes. After further treatment with a fixation/permeabilization solution (catalog number 420801, BioLegend), the cells were exposed to FITC-conjugated anti-APJ antibody, prepared using an FITC conjugation kit (Lightning-Link conjugation kit, Abcam) and unlabeled anti-APJ antibody (Cat. No. 20341-1-AP, Proteintech, CA, USA) for 30 min at 4 °C. After washing in wash buffer twice, the labeled cells were used for flow cytometry. The procedure involved acquiring 50,000 total events using a BD FACSVerse system (BD Biosciences, San Jose, CA, USA) and analysis of the findings using FlowJo v10.7 (Tree Star, Ashland, OR, USA). Negative gates were set based on the isotype control.

### 2.5. Muscle Cell Culture

To extract mononuclear cells from the quadriceps femoris of young and old mice (*n* = 5), tissue samples were treated with a 20 mL solution of 0.1% type I collagenase for 60 min at 37 °C. The harvested cells (1 × 10^4^ cells/cm^2^) containing heterogenous populations were cultured in α-minimal essential media (Gibco Life Technologies, Carlsbad, CA, USA) + 10% fetal bovine serum (Gibco Life Technologies; lot no. 42Q0170K) in six-well plates for 7 days. The cultured cells were then exposed to 0 ng/mL (control: culture medium only), 2.5 ng/mL mouse TNF-α, or 25 ng/mL TNF-α for 8 or 24 h. The gene expression of *Apelin* and *Apj* in muscle-derived cells was determined using real-time PCR in the same manner as that described above.

### 2.6. Statistics

Statistical analyses were performed using SPSS version 28.0.0.0 (190) (IBM, Armonk, NY, USA). The Shapiro–Wilk test was used to test for normality, and Levene’s test for the homogeneity of the variance. Mann–Whitney U tests were used to compare body weight and muscle mass between the two age groups. The unpaired t-test was used to compare muscle mass/body weight between the two age groups. As the gene and protein expression data were not normally distributed, the Mann–Whitney U test was used to compare gene and protein expression between the two age groups. Two-way ANOVA with the Bonferroni post-hoc test was used to compare the gene expression among control, 2.5 ng/mL TNF-α-, and 25 ng/mL TNF-α-stimulated cells. *p* < 0.05 was considered significant. All values are expressed as the mean ± standard deviation (SD).

## 3. Results

### 3.1. Muscle Mass of the Quadriceps of Young and Old Mice

**Figure 1:**
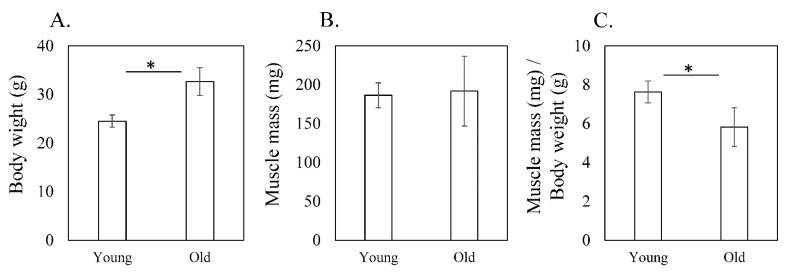
Muscle mass of the quadriceps of young and old mice. (**A**) Body weight (g), (**B**) muscle mass (mg), and (**C**) muscle mass (mg)/body weight (g) in young (3-month-old) and old (24-month-old) mice. Body weight was significantly higher in old mice than in young mice (*p* < 0.001; (**A**)). Muscle mass did not significantly differ between young and old mice (*p* = 0.481; (**B**)). However, muscle mass/body weight was significantly lower in old mice than in young mice (*p* < 0.001; (**C**)). Data are expressed as the mean ± standard deviation (SD). Asterisks indicate *p* < 0.05.

Body weight was significantly higher in old mice (32.6 ± 2.9 g) than in young mice (24.5 ± 1.3 g, *p* < 0.001; Figure 1A). Muscle mass did not significantly differ between young (186.5 ± 16.1 mg) and old mice (191.9 ± 45.0 mg) (*p* = 0.481; Figure 1B). However, muscle mass/body weight was significantly lower in old mice (5.8 ± 1.0 mg/g) than in young mice (7.6 ± 0.6 mg/g, *p* < 0.001; Figure 1C).

### 3.2. Expression of Apelin and APJ in the Quadriceps of Young and Old Mice

**Figure 2:**
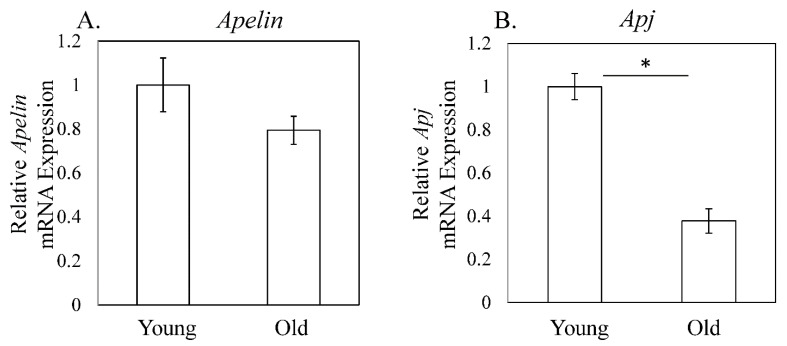
Relative expression of *Apelin,* and *Apj* mRNA in the quadriceps femoris. (**A**) *Apelin* and (**B**) *APJ* expression in young (3-month-old) and old (24-month-old) mice. There was no difference in Apelin mRNA expression between young and old mice (Figure 1A). *Apj* mRNA expression was significantly lower in old mice than in young mice (*p* < 0.001, Figure 1B). Data are expressed as the mean ± standard deviation (SD). Asterisks indicate *p* < 0.05.

**Figure 3:**
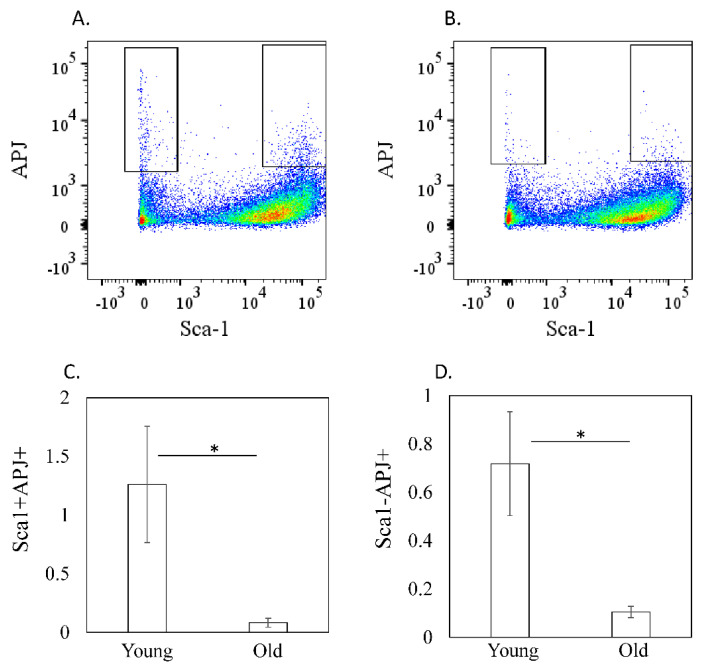
APJ−positive cells in the quadriceps femoris. (**A**,**B**) Dot plot analysis of CD45-negative myogenic cells. *X*-axis, Sca-1; *Y*-axis, APJ. (**C**,**D**) Ratio of APJ-positive cells among CD45-negative/Sca1-negative cells © and CD45-negative/Sca1-positive cells (**D**) in young (3-month-old) and old (24-month-old) mice. Significant reduction in APJ-positive cells was found in Sca1+ (*p* = 0.016) and Sca1− cells (*p* = 0.016). Data are expressed as the mean ± standard deviation (SD). Asterisks indicate *p* < 0.05 (Mann–Whitney U test).

*Apelin* expression was not significantly different (1.00 ± 0.12 (young) vs. 0.79 ± 0.06 (old), *p* = 0.253, Figure 2A), whereas *Apj* was significantly decreased in old compared to young mice (1.00 ± 0.06 (young) vs.0.38 ± 0.06 (Old), *p* < 0.001, Figure 2B). Further, the ratio of APJ-positive cells was also reduced in old mice among both Sca1-positive and Sca1-negative cells (Sca1-positive: 1.26 ± 0.50 (young) vs. 0.08 ± 0.04 (old), *p* = 0.016, Sca1-negative: 0.72 ± 0.22 (young) vs. 0.11 ± 0.02 (old), *p* = 0.016, Figure 3A,B).

### 3.3. Expression of TNF-α in the Quadriceps of Young and Old Mice

**Figure 4:**
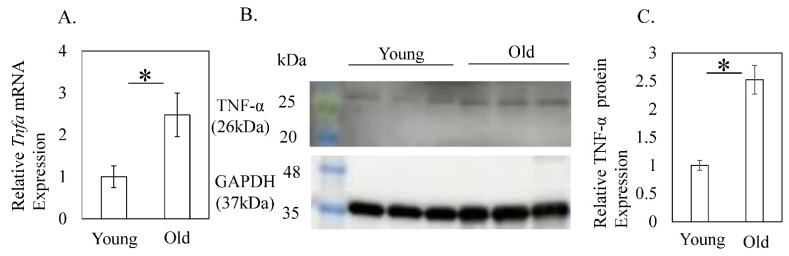
*Tnfa* mRNA and TNF-α protein expression in the quadriceps femoris. (**A**) *Tnfa* mRNA levels in young (3-month-old) and old (24-month-old) mice. (**B**) Image of a Western blot showing TNF-α protein expression relative to GAPDH at three (3M) and 24 months (24M). (**C**) TNF-α protein levels in the quadriceps femoris of young (3-month-old) and old (24-month-old) mice. Significant elevation of TNF-α mRNA and protein levels was found in the old group. Data are expressed as the mean ± standard deviation (SD). Asterisks indicate *p* < 0.05.

mRNA expression of *Tnfa* was significantly elevated in old compared to young mice (1.00 ± 0.26 (young) vs. 2.47 ± 0.52 (old), *p* = 0.034, Figure 4A). Western blotting showed that protein expression of TNF-α was likewise increased in old mice (1.00 ± 0.09 (young) vs. 2.52 ± 0.26 (old), *p* = 0.006, Figure 4B).

### 3.4. Effect of TNF-α on Apj Expression

**Figure 5:**
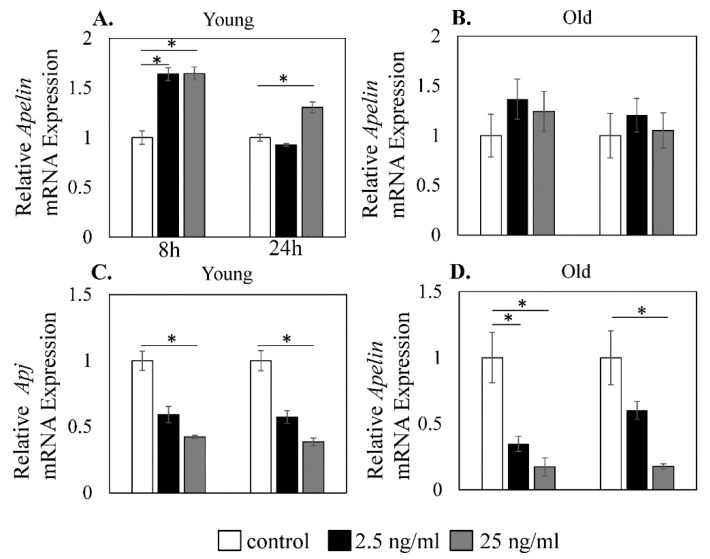
Effect of TNF-α on *Apelin* and *Apj* expression. Relative expression of *Apelin* (**A**,**B**) and *Apj* (**C**,**D**) in young and old mice-derived muscle cells after stimulation with 0 (control), 2.5, or 25 ng/mL mouse recombinant TNF-α for 8 and 24 h. Significant elevation of *Apelin* mRNA expression was only observed in young mice-derived muscle cells after TNF-α stimulation for 8 and 24 h (**A**,**B**). *Apj* mRNA expression was significantly reduced following TNF-α stimulation for 8 and 24 h in both young and old mice-derived muscle cells (**C**,**D**). Data are expressed as the mean ± standard deviation (SD). Asterisks indicate *p* < 0.05.

As our findings indicated that the expression level of APJ differed between young and old mice, we next evaluated whether the response of muscle cells to TNF-α was also different between the two age groups. Relative *Apelin* mRNA expression in young mice-derived cells is shown in Figure 5A, namely, control (8 h, 1.00 ± 0.07; 24 h, 1.00 ± 0.08), 2.5 ng/mL TNF-α (8 h, 1.64 ± 0.06; 24 h, 0.93 ± 0.01), and 25 ng/mL TNF-α (8 h, 1.64 ± 0.07; 24 h, 1.31 ± 0.01).

TNF-α significantly increased Apelin mRNA expression in young mice-derived muscle cells for 8 h (control vs. 2.5 ng/mL TNF-α: *p* < 0.001, control vs. 25 ng/mL TNF-α: *p* < 0.001, Figure 4A) and for 24 h (control vs. 25 ng/mL TNF-α: *p* < 0.001; Figure 5A).

Relative *Apelin* mRNA expression in old mice-derived cells is shown in Figure 5B, namely, control (8 h, 1.00 ± 0.21, 24 h, 1.00 ± 0.22), 2.5 ng/mL TNF-α (8 h, 1.37 ± 0.20; 24 h, 1.21 ± 0.17), and 25 ng/mL TNF-α (8 h, 1.24 ± 0.20; 24 h, 1.05 ± 0.18). No significant increase was observed in old mice-derived muscle cells (Figure 5B).

Relative *Apj* mRNA expression in young mice-derived cells is shown in Figure 5C, as control (8 h, 1.00 ± 0.07; 24 h, 1.00 ± 0.08), 2.5 ng/mL TNF-α (8 h, 0.59 ± 0.06; 24 h, 0.57 ± 0.05), and 25 ng/mL TNF-α (8 h, 0.42 ± 0.01; 24 h, 0.39 ± 0.03). Relative *Apj* mRNA expression in old mice-derived cells is shown in Figure 5D, as control (8 h, 1.00 ± 0.19, 24 h, 1.00 ± 0.20), 2.5 ng/mL TNF-α (8 h, 0.35 ± 0.06; 24 h, 0.60 ± 0.07), and 25 ng/mL TNF-α (8 h, 0.17 ± 002; 24 h, 0.18 ± 0.03). *Apj* was significantly reduced in both young and old mice-derived muscle cells following stimulation with exogenous TNF-α for 8 h (young, control vs. 25 ng/mL TNF-α, *p* < 0.001; old, control vs. 2.5 ng/mL TNF-α: *p* = 0.012, control vs. 25 ng/mL TNF-α: *p* = 0.009, Figure 4B) and 24 h (young, control vs. 25 ng/mL TNF-α, *p* < 0.001; old, control vs. 25 ng/mL TNF-α: *p* = 0.007, Figure 5C,D).

## 4. Discussion

The purpose of this study was to examine interconnection between TNF-α elevation and APJ reduction with aging. We showed that old mice had reduced *Apj* expression and a reduced ratio of APJ-positive cells compared to young mice. In contrast, they had significantly higher concentrations of TNF-α than young mice. Further, exposing muscle-derived cells to exogenous TNF-α caused *Apj* mRNA expression to significantly decrease. Together, our results suggest that the reduction in APJ in old mice may be associated with increased TNF-α.

Sarcopenia has been extensively studied using mouse models. Mice have a lifespan of 2–3 years [32]. The ratio of muscle/body weight has been proposed to be a useful sarcopenia index in rodent [33]. A previous study reported that loss of muscle mass (muscle weight/body weight) first becomes evident in 24-month-old C57BL/6J mice [31]. Consistent with a previous study [31], a lower muscle weight/body weight was observed in old mice (24-month-old) compared to young mice. Therefore, we used 24-month-old mice as an aged model. Sarcopenia is defined by low levels of measures for three parameters: (1) muscle strength, (2) muscle quantity/quality and (3) physical performance as an indicator of severity [6]. In our study, we did not assess muscle strength, quality, or physical performance. In addition, muscle mass did not differ between young and old mice. Therefore, the mice used in this study may be insufficient as a sarcopenia model.

APJ has been previously reported to be associated with age-related muscle atrophy [11]. Pax7-expressing muscle stem cells express APJ, and the number these cells is reduced with age. In contrast, apelin stimulates glucose uptake and Akt phosphorylation in myotubes, suggesting that mature myogenic cells also express APJ. Previous studies have implicated Sca1 as a regulator of differentiation in myogenic cells [34]. While myoblasts are negative for Sca1, mRNA expression increases upon myogenic differentiation. In our study, we observed APJ-positive cells among both Sca1-positive and Sca1-negative cells, and that their ratio decreased in old mice. Our results thus suggest that reduced APJ expression with age reflects decreased expression in both immature and mature myogenic populations.

Several studies have shown that TNF-α rises with age in mice and humans [20,27,35]. Plasma TNF-α protein level increases with age [27]. TNF-α mRNA and protein levels are elevated in the SM of frail elderly compared to healthy young men and women [35]. Real-time PCR and flow cytometric analysis has shown that TNF-α mRNA expression in immune cells and TNF-α protein-positive macrophages are increased in skeletal muscle of old mice [20]. Similar to a previous report [20], we confirmed that TNF-α mRNA and protein expression in SM was significantly elevated in old compared to young mice. Stimulation with TNF-α significantly reduced APJ expression in muscle-derived cells from young and old mice compared to vehicle control cells. The reduced APJ expression in old mice may be associated with elevated TNF-α levels.

A previous study reported that TNF-α stimulated apelin expression in mice and human adipose tissue [29]. Consistent with this report [29], TNF-α also stimulated *Apelin* mRNA expression in young mice-derived muscle cells. However, TNF-α failed to stimulate apelin expression in old mice-derived muscle cells. Apelin ameliorates TNF-α mediated physiological changes in hepatocytes [28], suggesting that apelin exhibits an anti-inflammatory role toward TNF-α-induced inflammation. Lack of negative feedback by apelin may result in an elevation of inflammatory state by the TNF-a of muscle in old mice. Differences in the response of young and aged cells to inflammatory stimuli have been reported [36,37]. For example, adipocytes from old mice produce more IL-6, TNF-α, and PGE2 than those from young mice [37]. In addition, differentiation conditions could also alter cytokine response [38]. The muscle-derived used cells in the present study represented a heterogenous population and the proportion of differentiated/undifferentiated cells differed between young and old mice cells. Changes in cell phenotype with aging or a different proportion of cell populations may be associated with the different response to TNF-α in apelin expression between young and old-derived muscle cells.

There were several limitations in this study. First, only two time points were investigated. To better understand the pathogenesis of sarcopenia, further studies should analyze changes across a greater number of time points. Second, we only used muscle mass as an indicator of the pathogenesis of sarcopenia and were unable to examine the pathology of the tissue. Finally, it remains unclear whether TNF-α directly regulates APJ expression.

## 5. Conclusions

TNF-α suppresses APJ expression in muscle cells in vitro. The increase in TNF-α observed in SM with age may induce a decrease in APJ expression.

## Figures and Tables

**Table 1:** Primer sequences.

Gene	Direction	Primer Sequence (5′–3′)	Product Size (bp)
*Apelin*	F	TGA ATC TGA GGC TCT GCG TG	223
	R	ATG GGG CCC TTA TGG GAG AG	
*Apj*	F	TAC GCC AGT GTC TTT TGC CT	159
	R	CAC CAT GAC AGG CAC AGC TA	
*Tnfa*	F	CTG AAC TTC GGG GTG ATC GG	122
	R	GGC TTG TCA CTC GAA TTT TGA GA	
*GAPDH*	F	AAC TTT GGC ATT GTG GAA GG	223
	R	ACA CATT GGG GGT AGG AAC A	

## Data Availability

The data presented in this study are available on request from the corresponding author.
